# Exploring Groin Hernias: A Visual 3D Guide to Diagnosis and Treatment

**DOI:** 10.3389/jaws.2024.13642

**Published:** 2024-09-10

**Authors:** M. Miserez, S. Van Hoef

**Affiliations:** ^1^ Department of Abdominal Surgery, University Hospital Gasthuisberg, KU Leuven, Leuven, Belgium; ^2^ Department of Abdominal Surgery, Jessa Hospital—Sint Trudo Hospital, Sint-Truiden, Belgium

**Keywords:** inguinal hernia, femoral hernia, diagnosis, repair, 3D video

## Introduction

Inguinal hernia repair is one of the most commonly performed procedures, with roughly more than 20 million procedures performed annually worldwide [1]. This amount is easy to understand when considering the high lifetime risk of inguinal hernia development, which is 3% and 27% in women and men respectively [1]. Femoral hernias on the other hand are 4 times more common in female patients [1]. Several different methods for repair exist: open versus minimally invasive and mesh versus non-mesh. While originally described as an open pure tissue repair, the use of prostheses and their improved long-term outcomes in preventing hernia recurrences have led many surgeons to move away from traditional tissue-based repairs, as illustrated by the Danish Hernia Database, showing almost a 100% mesh usage for inguinal hernia repair [1–4]. Mesh can be placed in 2 different planes, the anterior and posterior plane. While the anterior plane can only be accessed through an open approach, first coined by Lichtenstein in 1989 [5], the posterior plane can be accessed through both an open and laparo-endoscopic approach.

As recurrence declined, the focus shifted towards avoiding chronic post-operative pain, which affects about 10%–12% of patients at 3 months follow-up, with a 0.5%–6% risk of pain affecting everyday life at 1 year [6].

Current guidelines recommend a mesh-based repair technique for the majority of patients undergoing inguinal hernia repair. First choice is a posterior laparo-endoscopic approach provided that a surgeon with specific expertise and sufficient resources is available [1]. However, there are patient and hernia characteristics that warrant Lichtenstein as first choice [1]. Large surveys, as well as registry-data are in line with this and show a predominant use of a laparoscopic TEP or TAPP-approach or an open anterior Lichtenstein approach [3, 4, 7–9]. Although the evidence for a tissue-based repair is (very) low, the Shouldice technique can be suggested after careful patient selection in a minority of patients and if expertise is available [1].

Shared decision-making between surgeon and patient will take into account patient related factors, patient opinion, hernia characteristics, surgeon’s preference and local access to resources. For patients to be well informed in the preoperative setting, it is useful to learn about the difference between an inguinal and femoral hernia, anterior vs. posterior mesh placement, and the course of the inguinal nerves. It will allow them to better understand both the surgical procedure and the risks of recurrence and chronic pain. However, illustrating the surgical anatomy of the groin in a simplified yet correct and attractive way remains problematic. Therefore, we developed a video which will hopefully serve as an easy-to-understand guide of the groin and explains the normal anatomy, the pathophysiology of an inguinal hernia and the concept of open Lichtenstein and posterior (laparoscopic) repair. We believe this video will also be very useful for teaching of students and surgeons in training, in order to better understand the difficult anatomy of the groin and the most popular options for mesh repair of groin hernias, in line with recently published guidelines [2].

## Result

A 3D-reconstruction of the right male groin was made. It provides a clear overview of the surgical anatomy, showing both an anterior, posterior, sagittal and axial view of the groin, highlighting the most important structures. We continue by illustrating the pathophysiology of both a direct, indirect and femoral hernia. This is further supported by a clinical image showing an inguinal hernia, during rest and Valsalva. The role and types of meshes used are shown, displaying their distinct properties. Finally we end by illustrating both the anterior and posterior approach to inguinal hernia repair, hereby highlighting key anatomical structures relevant for post-operative outcome. The full video is not more than 6 min in length, is rendered in high definition, and available in English/Dutch audio and subtitles. The video will be available on YouTube.

## Discussion

Inguinal hernia repair is one of the most frequently performed procedures worldwide. A basic knowledge of the anatomy is indispensable for patients. However, illustrating this in a comprehensible matter remains problematic. This video provides a 3D-reconstruction of the male groin, and explains the normal anatomy, the pathophysiology of an inguinal hernia and the concept of open Lichtenstein and posterior (laparoscopic) repair. It is accompanied by images, showing the typical clinical presentation and different mesh-types. We hope this video will improve surgical training for groin hernia repair and facilitate the shared decision-making process preoperatively between patient and surgeon globally.
